# Comparison of prognosis in two methods for the lingual nerve repair: direct suture with vein graft cuff and collagen allograft method

**DOI:** 10.1186/s40902-022-00335-9

**Published:** 2022-03-01

**Authors:** Shigeyuki Fujita, Itaru Tojyo, Takashi Nakanishi, Shigeru Suzuki

**Affiliations:** Oral and Maxillofacial Surgery, Wakayama Medical University, Kimiidera 811-1, Wakayama, 641-8509 Japan

**Keywords:** Lingual nerve, Collagen allograft, Direct perineurial repair, Third molar extraction, Microneurosurgery

## Abstract

**Background:**

No studies have compared the outcomes of direct perineurial suture with vein graft cuff repair and indirect collagen allograft repair of the lingual nerve following an injury. Therefore, we evaluated and compared the outcomes of each over a 1-year observation period. We retrospectively assessed 20 patients who had undergone microneurosurgical repair of unilateral lingual nerve injuries at the Wakayama Medical University Hospital between May 2015 and March 2019. We utilized two different methods for lingual nerve repair, i.e., direct perineurial repair with a vein graft cuff and interpositional collagen allograft repair. Sensory and taste function in the lingual nerve were preoperatively evaluated using a static two-point discrimination test, superficial pain/tactile sensation test, tests for the pressure pain threshold (Semmens-Weinstein monofilament), test for thermal discrimination hot and cold sensation, and a taste discrimination test. These tests were performed again at 6 and 12 months postoperatively.

**Results:**

Compared to the preoperative conditions, all patients showed improved sensory reactions. Functional sensory recovery outcomes were defined by Pogrel’s criteria, Medical Research Council Scale grades, and functional sensory recovery. In each group, all patients improved after the operation. However, the operation time was significantly shorter for an interpositional collagen allograft repair as compared to that for a direct perineurial repair with a vein graft cuff.

**Conclusions:**

There were no statistically significant differences between the two repair Methods, except for the operation time. Both methods led to satisfactory results for all criteria. From an economic point of view, direct perineurial repair with a vein graft cuff is meaningful; however, the esthetic effect on the donor site should be considered*.* Conversely, interpositional collagen allograft repair has the advantage of a greatly shortened operation time.

## Background

Wisdom tooth extraction is an important procedure in dentistry. Unfortunately, lingual nerve(LN)damage, though rare, occurs after the extraction of the lower third molar. occurs seldom after the lower third molar extraction. A mild LN injury will heal without any treatment. However, a nerve repair is essential in case of severe LN injuries and performed using various methods [1–4]. Compared with an injured inferior alveolar nerve, an injured LN is considered to be more difficult to repair [5, 6]. No universal standard guidelines for when and how to perform LN repairs have been established yet [7–9]. Over the past 20 years, we have performed several LN repairs by direct anastomosis with a vein graft cuff in cases with severe tongue neuropathy, we have already reported an outline of this procedure [10]. We performed a retrospective cohort study comparing the functional recovery between patients who received a direct perineurial nerve anastomosis with a vein graft cuff (group A) and patients who received an artificial nerve repair (group B). Because there have been no such reports today, we have reported the detailed findings of these procedures.

## Methods

**Fig. 1:**
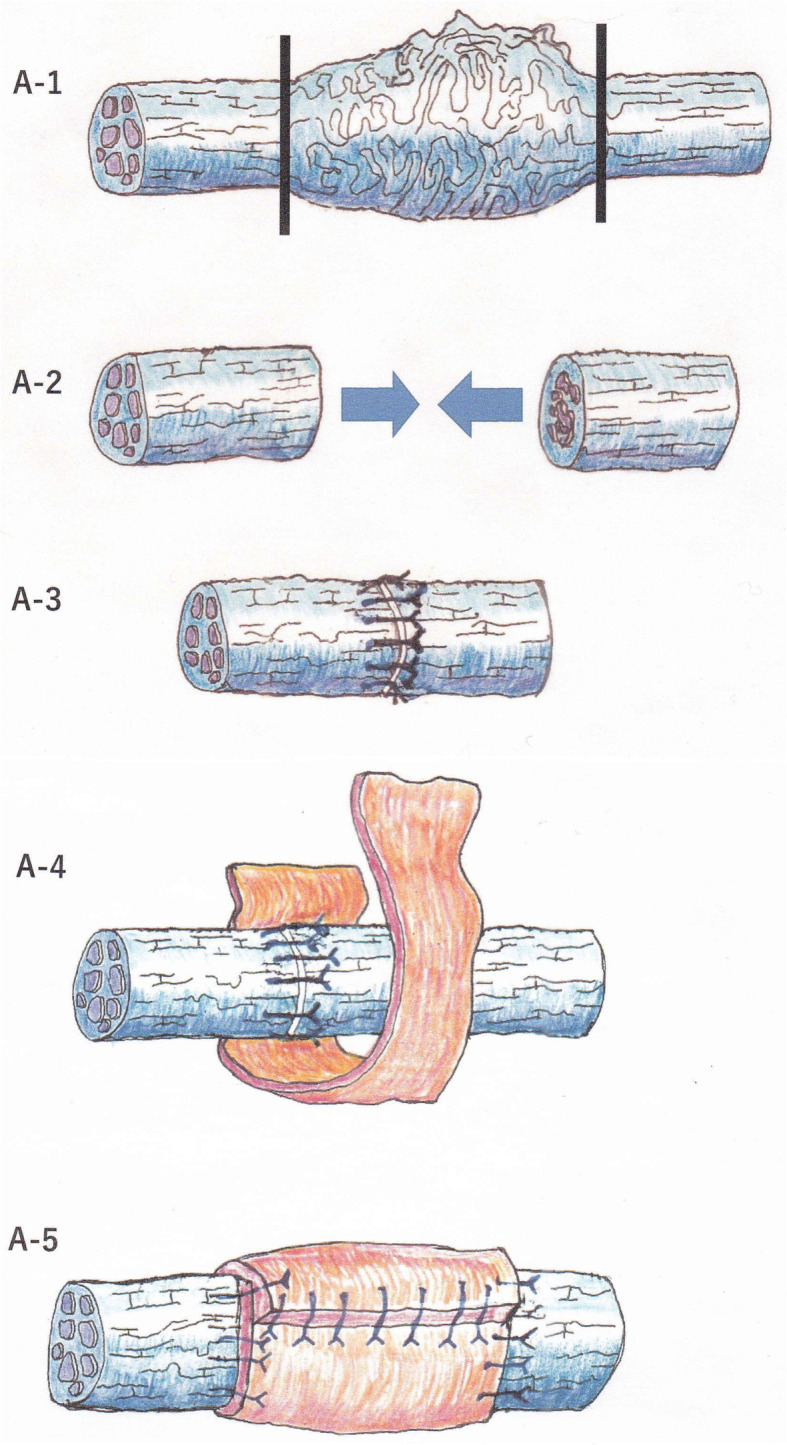
Direct perineurial repair with a vein graft cuff. A-1:Nerve stump preparation. Neuroma resection at the clinical margin of the neuroma fails to complete nerve preparation. A-2:Remove as much scar tissue as possible from the torn nerve, mobilized and trimmed to a point where the fascicles could be identified in a microsurgical field. A-3:Direct end-to-end epineural nerve suture without tension was performed. A-4: A segment of the external jugular vein was tagged for use as a cuff to cover the sutured nerve. The autogenous vein graft was split longitudinally and turned inside-out. A-5: The vein graft encased the sutured site, and attached to the epineural membrane with 8-0 nylon on each side

**Fig. 2:**
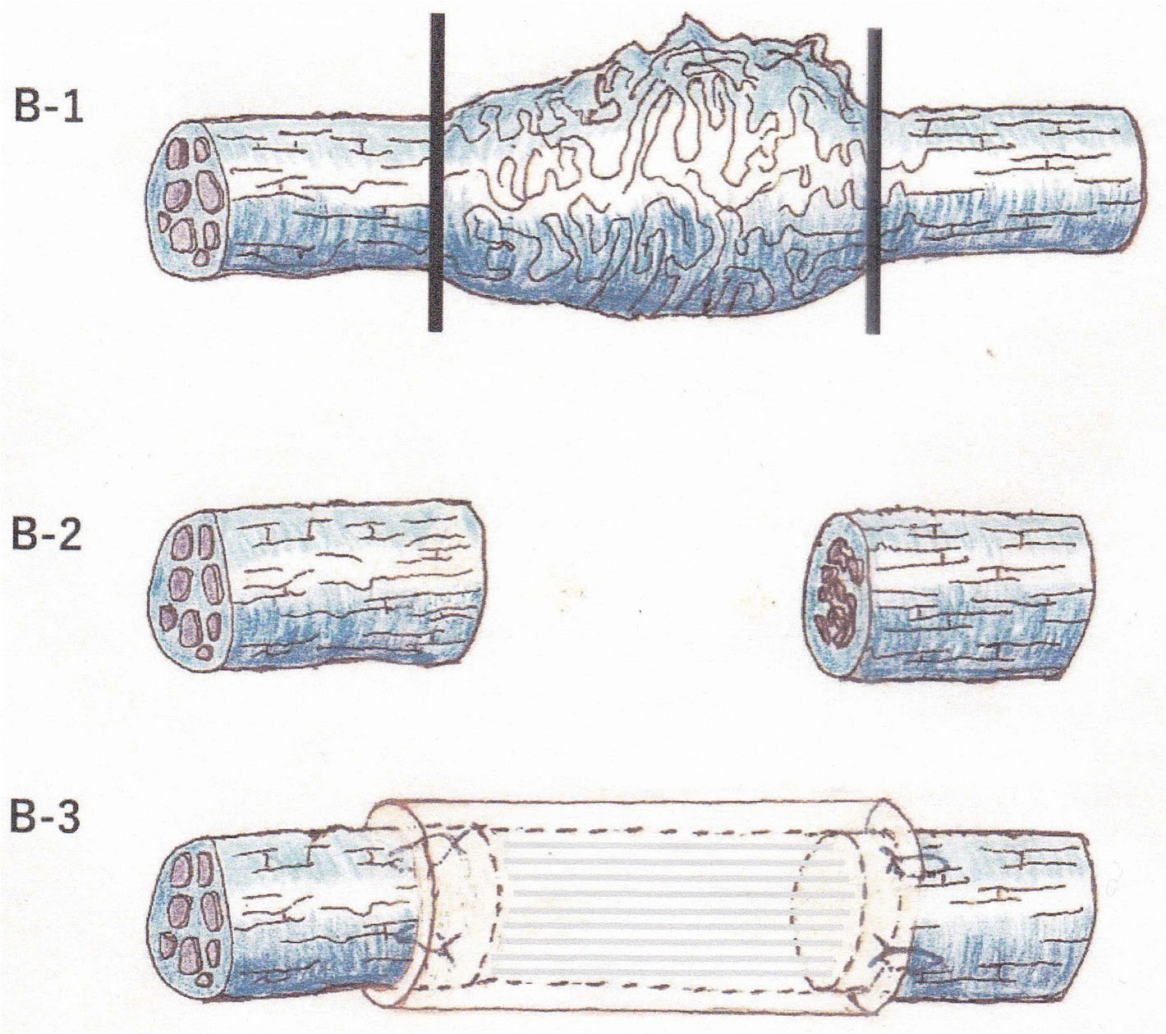
Interpositional collagen allograft repair. B-1:Nerve stump preparation. Neuroma resection at the clinical margin of the neuroma fails to complete nerve preparation. B-2:After complete excision of the injured and degenerated nerve, a healthy bilateral stump surface is revealed. B-3:An artificial collagen nerve graft, Renerve^Ⓡ^, which is several millimeters longer than the nerve gap, is inserted between ends of healthy nerve stumps and sutured with 8-0 nylon

For this 1-year observational study, observational data were collected from patients who underwent microneurosurgery for a unilateral LN injury caused during third molar extraction at the Wakayama Medical University Hospital between May 2014 and March 2019; this was also the only inclusion criterion of the study. Written informed consent was obtained from all patients before the surgery. For each patient, the microneurosurgical LN repair was performed under general anesthesia by the same surgeon (S. F.). Both groups had similar background characteristics, these patients had no serious conditions, except for LN disorder. The following two types of torn LN microsurgical repairs were performed: direct perineurial suturing with a vein graft cuff (group A, *n* = 10) and indirect interpositional collagen allograft repair using RENERVE® (group B, *n* = 10). The following neurosensory assessments were performed preoperatively and at 6 and 12 months after the microsurgery. Brushstroke directional sensation with a camel hair brush (Brush); horizontal, vertical, and rotational stimulating movements were applied, (scores:0 means recognized not at all,1 means recognized only 1 direction, 2 means recognized 2 directions and 3 means recognized all movements). Static 2-point discrimination (2PD), pressure pain threshold: the Semmens-Weinstein monofilament (SWM); which is composed of 20 different diameter monofilaments was used. One was assigned to the smallest-diameter monofilament, and 20 was the largest-diameter monofilament. Thermal discrimination (Thermal) hot and cold sensations; hot water (42 °C): cold sensation; fragment of ice (0 °C), Sharp touch with needle (Pin prick) and Gustatory sensation assessed with localized testing discs (Sanwa Kagaku Kenkyusho, Japan); salty, sodium chloride (1mol/L); Sweet, sucrose (1 mol/L); sour, acetic acid (0.4mol/L); and bitter, quinine (0.1 mol/L). Microneurosurgery procedures for LN injury were performed in all cases as previously reported [10]. Briefly, the LN was exposed through an intraoral mucosal incision and lingual flap reflection. The standard subperiosteal approach to the LN was used to identify the proximal and distal nerve segments, the surgeon then worked toward the site of the injury. The neuroma and peripheral scar surrounding the torn nerve was removed; after this procedure, both nerve ends could touch without tension. Thereafter, using 8-0 nylon sutures, either direct end-to-end nerve suturing with vein graft cuff repair or interpositional collagen allograft nerve suturing repair was performed (Figs. 1 and 2). Optical magnifying glasses (250 mm), and an operating microscope (Superlux 301, Zeiss, Germany) were used during the surgery. All data were statistically analyzed for significance using JMP Pro 12 (SAS Institute Inc. Cary, NC, USA). The Mann-Whitney test or the Fisher’s exact test were used to compare the postsurgical outcomes (defined using Pogrel’s criteria), Sunderland grades, Medical Research Council Scale (MRCS) grades, and functional sensory recovery (FSR) at each stage (preoperative, 6 months postoperatively, and 12 months postoperatively) between groups A and B. For all analyses, the statistical significance was set at *P* < 0.05. Objective data on neurosensory recovery were correlated with the MRCS grades; grades S3, S3+, and S4 indicated the presence of FSR. Grade S3 corresponded to a return of some superficial pain and tactile sensation without an overresponse and a 2-points discriminations of over 15 mm. This study was performed in accordance with the Declaration of Helsinki and was approved by the Wakayama Medical University Institutional Review Board (No. 1689).

## Results

**Fig. 3:**
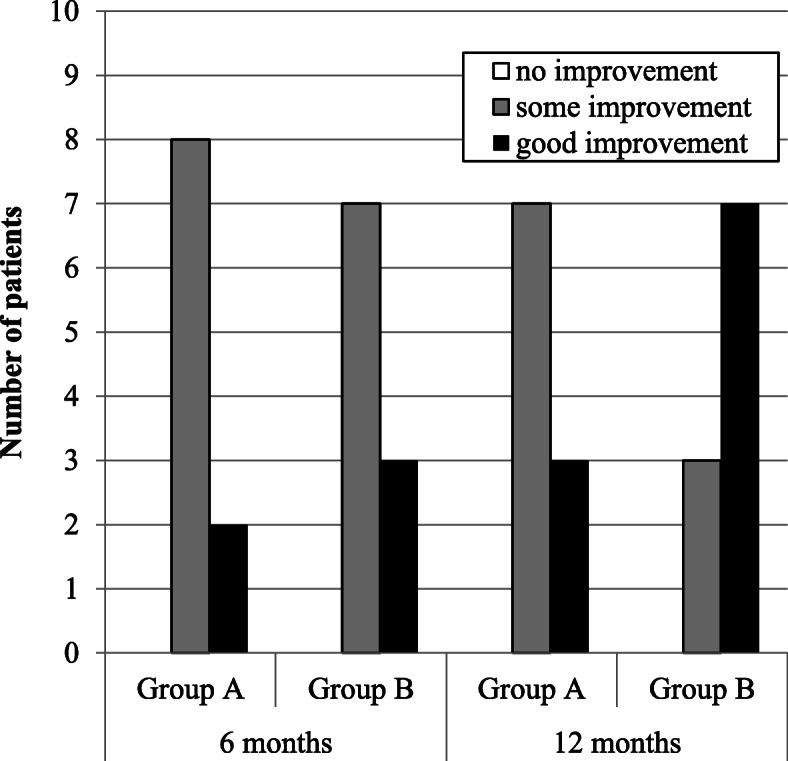
Postsurgical outcomes classified by the criteria of Pogrel

**Fig. 4:**
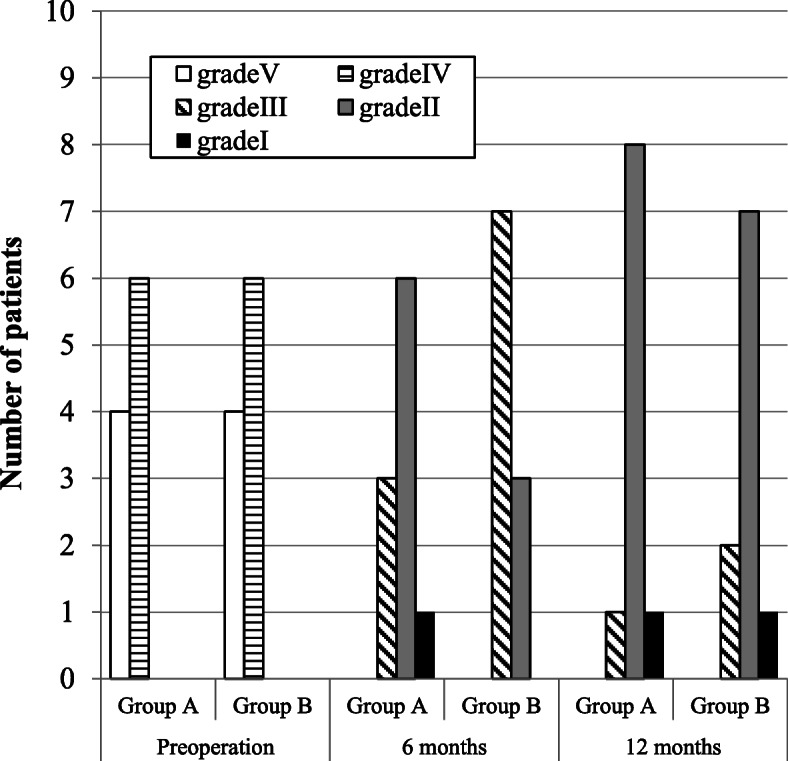
Sunderland grades

**Fig. 5:**
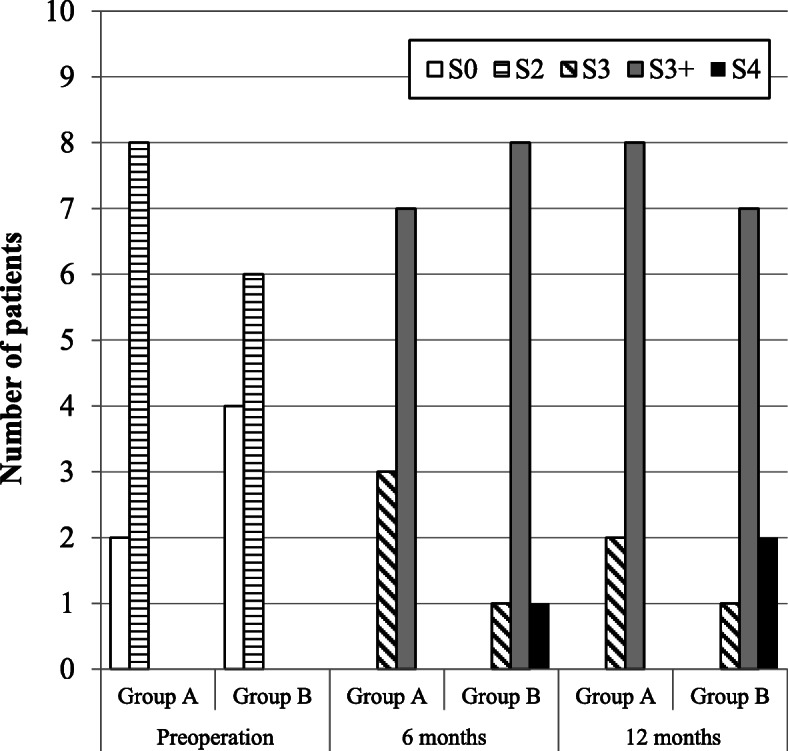
MRCS grades

Among the 20 patients included, 13 were women (65%) and 7 were men (35%). The mean age of all 20 patients was 35.8 years (range: 21–53 years), while the median ages in groups A and B were 37.4 and 34.3 years, respectively. For all 20 patients, the median interval between nerve injury and repair was 17.95 months (range: 4–96 months): however, the median intervals in groups A and B were 19.9, and 16.0 months, respectively. Detailed pre-and postoperative data of each group are presented in Tables 1, 2, 3, 4, 5, 6, and 7. The postsurgical outcomes were classified using the criteria given by Pogrel [11]. Improvement was determined to be good if two of the following three criteria were met: (i) an improvement of 5 or more on von Frey’s hair test, (ii) an improvement of 10 mm or more in 2PD, and (iii) an improvement from no sensation to the ability to detect hot and cold water, or from the ability to detect hot and cold water to the ability to differentiate between three or less Minnesota thermal discs. Some improvement was confirmed if any two of the following were achieved: (i) an improvement of 2–5 in von Frey’s hair test, (ii) an improvement of 5–10 mm in 2PD, and (iii) an improvement in temperature sensation. No improvement was deemed to have occurred if any two of the following were recorded: (i) an improvement or a decrease of up to 2 hairs in either direction in von Frey’s hair test, (ii) a change of less than 5 mm in either direction in 2PD, and (iii) no improvement in temperature sensation. The patient’s condition was determined to have worsened after microneurosurgery if at least one of the following was recorded: (i) a decrease of more than 2 hairs in von Frey’s hair test, (ii) an increase of 5 mm or greater in 2PD, and (iii) decreased temperature sensation from the preoperative examination. The Pogrel’s criteria was summarized in Table 8. According to the criteria, 6 months after the operation, two patients exhibited a good improvement, and eight patients exhibited some improvement in group A. Conversely, three patients exhibited a good improvement and seven patients exhibited some improvement in group B. One year after the operation, three patients exhibited a good improvement and seven patients exhibited some improvement in group A. Similarly, seven patients exhibited a good improvement and three patients exhibited some improvement in group B. These intergroup differences were not significant (Table 9 and Fig. 3). For the initial nerve injury, the Sunderland grades were V in four patients in groups A and B and VI in six patients in groups A and B. Six months postoperatively, the Sunderland grades recovered to III, II, and I in three, six, and one patient in group A, respectively, and to III and II seven and three patients in group B, respectively. Twelve months postoperatively, the grades improved to III, II, and I in one, eight, and one patient in group A, respectively, and to III, II, and I in two, seven, and one patient in group B, respectively. These intergroup differences were not significant (Table 10 and Fig. 4). In group A, the MRCS grades of the initial nerve injury were S0 and S2 in two and eight patients, respectively. Conversely, in group B, the MRCS grades of the initial nerve injury were S0 and S2 in four and six patients, respectively. At 6 months postoperatively, the MRCS grades were S3 and S3+ in three and seven patients in group A, respectively. In group B, the MRCS grades were S3, S3+, and S4 in one, eight, and one patient, respectively. At 12 months postoperatively, the MRCS grades were S3 and S3+ in two and eight patients in group A, respectively. In group B, the MRCS grades were S3, S3+, and S4 in one, seven, and two patients, respectively. These intergroup differences were not significant (Table 11 and Fig. 5). The objective functional sensory results, defined by MRCS grades of S3, S3+, and S4, were already accomplished in both groups at 6 months after the LN repair. The FSR was also filled in all cases in both groups after 6 months of LN repair, which was not significantly different between the two groups. Other variables, including age, interval from injury to repair, and initial Sunderland grade of injury, did not differ significantly between the two groups. Detailed statistically analyzed data are presented in Tables 7, 9, 10, and 11 and Figs. 3, 4, and 5.

**Table 1:** Preoperative data and the operation time for group A (patients underwent a direct suturing repair)

No.	Age (years)	Gender	Time from injury to repair (months)	Brush	2PD (mm)	SWM	Themal (hot/cold)	Pin prick	Sunderland grade	MRCS	Gustatory sensation	FSR	Operation time (minutes)	
**1**	**22**	**M**	**4**	**0**	**> 20 (5)**	**10 (1)**	**Response/response**	**No response**	**VI**	**S2**	**No**	**−**	**542**	
**2**	**34**	**M**	**4**	**0**	**> 20 (5)**	**16 (2)**	**No response/no response**	**No response**	**V**	**S0**	**No**	**−**	**349**	
**3**	**36**	**M**	**4**	**0**	**> 20 (5)**	**10 (1)**	**No response/no response**	**No response**	**V**	**S0**	**No**	**−**	**607**	
**4**	**38**	**M**	**4**	**0**	**> 20 (5)**	**12 (1)**	**No response/no response**	**Weak**	**VI**	**S2**	**No**	**−**	**279**	
**5**	**47**	**F**	**5**	**1**	**> 20 (5)**	**7 (1)**	**No response/response**	**No response**	**VI**	**S2**	**No**	**−**	**362**	
**6**	**30**	**M**	**6**	**1**	**> 20 (5)**	**11 (2)**	**No response/response**	**No response**	**VI**	**S2**	**No**	**−**	**595**	
**7**	**34**	**F**	**7**	**1**	**> 20 (5)**	**4 (1)**	**No response/response**	**No response**	**VI**	**S2**	**No**	**−**	**417**	
**8**	**50**	**F**	**10**	**0**	**> 20 (5)**	**10 (1)**	**Allodynia/allodynia**	**No response**	**V**	**S2**	**No**	**−**	**379**	
**9**	**48**	**M**	**65**	**0**	**> 20 (5)**	**10 (1)**	**No response/response**	**No response**	**VI**	**S2**	**No**	**−**	**312**	
**10**	**35**	**F**	**90**	**0**	**> 20 (5)**	**12 (2)**	**Allodynia/allodynia**	**Weak**	**V**	**S2**	**No**	**−**	**327**	

Gender: F means female and M means male. Brush: Brushstroke directional sensation; horizontal, vertical, and rotational stimulating movement were applied (0 means recognized not at all, 1 means recognized only 1 direction, 2 means recognized 2 directions, and 3 means recognized all movements)

2PD: Static 2-point discrimination; the values in parentheses are the opposite and control values

SWM: The Semmens-Weinstein monofilament is composed of 20 different diameter monofilaments. 1 was assigned to the smallest-diameter and 20 was the largest-diameter monofilament. The values in parentheses are the opposite and control values

Thermal: Thermal discrimination is hot and cold sensations; hot water 42 °C: cold sensation; fragment of ice 0 °C

Pin prick: Sharp touch with needle. *MRCS* Medical research Council Scale. *FSR* functional sensory recovery

**Table 2:** Preoperative data and the operation time for group B(patients underwent an artificial nerve repair)

No.	Age (years)	Gender	Time from injury to repair (months)	Brush	2PD (mm)	SWM	Themal (hot/cold)	Pin prick	Sunderland grade	MRCS	Gustatory sensation	FRS	Operation time (minutes)	
**1**	**33**	**F**	**4**	**0**	**> 20 (5)**	**16 (2)**	**No response/no response**	**Weak**	**VI**	**S2**	**No**	**−**	**201**	
**2**	**36**	**F**	**5**	**0**	**> 20 (5)**	**16 (1)**	**No response/no response**	**No response**	**V**	**S0**	**No**	**−**	**401**	
**3**	**40**	**F**	**5**	**0**	**> 20 (5)**	**20 (1)**	**No response/no response**	**No response**	**V**	**S0**	**No**	**−**	**301**	
**4**	**42**	**F**	**5**	**0**	**> 20 (5)**	**19 (2)**	**No response/no response**	**No response**	**V**	**S0**	**No**	**−**	**201**	
**5**	**53**		**5**	**0**	**> 20 (5)**	**11 (1)**	**No response/allodynia**	**Weak**	**VI**	**S2**	**No**	**−**	**306**	
**6**	**24**	**F**	**6**	**0**	**> 20 (5)**	**14 (1)**	**No response/no response**	**Weak**	**VI**	**S2**	**No**	**−**	**232**	
**7**	**21**	**F**	**8**	**0**	**> 20 (5)**	**10 (1)**	**No response/no response**	**Weak**	**VI**	**S2**	**No**	**−**	**261**	
**8**	**30**	**F**	**8**	**0**	**> 20 (5)**	**14 (1)**	**No response/no response**	**No response**	**V**	**S0**	**No**	**−**	**401**	
**9**	**31**	**F**	**18**	**0**	**> 20 (5)**	**14 (2)**	**No response/response**	**Weak**	**VI**	**S2**	**No**	**−**	**310**	
**10**	**33**	**M**	**96**	**1**	**> 20 (5)**	**8 (1)**	**Allodynia/allodynia**	**No response**	**VI**	**S2**	**No**	**−**	**283**	

Gender: F means female and M means male. Brush: Brushstroke directional sensation, horizontal, vertical, and rotational stimulating movement were applied (0 means recognized not at all, 1 means recognized only 1 Direction, 2 means recognized 2 directions and 3 means recognized all movements)

2PD: Static 2-point discrimination; the values in parentheses are the opposite and control values

SWM: The Semmens-Weinstein monofilament is composed of 20 different diameter monofilaments. 1 was assigned to the smallest-diameter and 20 was the largest-diameter monofilament. The values in parentheses are the opposite and control values

Thermal: Thermal discrimination is hot and cold sensations; hot water 42 **°**C: cold sensation; fragment of ice 0 **°**C

Pin prick: Sharp touch with needle. *MRCS* Medical research Council Scale. *FSR* functional sensory recovery

**Table 3:** Postoperavive data of group A (recoded at 6 months)

No.	Age (years)	Gender	Time from injury to repair(months)	Brush	2PD (mm)	SWM	SWM (hot/cold)	Pin prick	Sunderland grade	MRCS	Gustatory sensation	FSR
**1**	**22**	**M**	**4**	**3**	**8**	**1 (1)**	**Response/response**	**Weak**	**I**	**S3+**	**Sweet**	+
**2**	**34**	**M**	**4**	**3**	**15**	**8 (1)**	**Response/response**	**Weak**	**II**	**S3+**	**No**	**+**
**3**	**36**	**M**	**4**	**3**	**13**	**8 (1)**	**Response/response**	**Clear**	**II**	**S3+**	**No**	**+**
**4**	**38**	**M**	**4**	**3**	**> 20**	**10 (1)**	**Response/response**	**Weak**	**III**	**S3**	**No**	**+**
**5**	**47**	**F**	**5**	**3**	**13**	**6 (1)**	**No response/response**	**Weak**	**III**	**S3+**	**No**	**+**
**6**	**30**	**M**	**6**	**3**	**14**	**6 (1)**	**Response/response**	**Weak**	**II**	**S3+**	**No**	**+**
**7**	**34**	**F**	**7**	**3**	**13**	**4 (1)**	**Response/response**	**Weak**	**II**	**S3+**	**No**	**+**
**8**	**50**	**F**	**10**	**3**	**8**	**5 (1)**	**Response/response**	**Clear**	**II**	**S3+**	**No**	**+**
**9**	**48**	**M**	**65**	**3**	**16**	**6 (1)**	**Response/response**	**Weak**	**II**	**S3**	**No**	**+**
**10**	**35**	**F**	**90**	**3**	**18**	**8 (1)**	**Response/response**	**Clear**	**III**	**S3**	**No**	**+**

Gender: F means female and M means male. Brush: Brushstroke directional sensation; horizontal, vertical, and rotational stimulating movement were applied (0 means recognized not at all, 1 means recognized only 1 direction, 2 means recognized 2 directions and 3 means recognized all movements)

2PD: Static 2-point discrimination; the values in parentheses are the opposite and control values

Thermal: Thermal discrimination is hot and cold sensations; hot water 42 °C: cold sensation; fragment of ice 0 °C

SWM: The Semmens-Weinstein monofilament is composed of 20 different diameter monofilaments. 1 was assigned to the smallest-diameter and 20 was the largest-diameter monofilament. The values in parentheses are the opposite and control values

Pin prick: Sharp touch with needle. *MRCS* Medical research Council Scale. *FSR* functional sensory recovery

**Table 4:** Postoperavive data of group B (recoded at 6 months)

No.	Gender	Age (years)	Time from injury to repair (months)	Brush	2PD (mm)	SWM	Themal (hot/cold)	Pin prick	Sunderland grade	MRCS	Gustatory sensation	FSR
**1**	**F**	**33**	**4**	**3**	**10**	**7 (1)**	**Response/response**	**weak**	**II**	**S3+**	**No**	**+**
**2**	**F**	**36**	**5**	**3**	**13**	**12 (1)**	**No response/response**	**Weak**	**III**	**S3+**	**Salt**	**+**
**3**	**F**	**40**	**5**	**3**	**10**	**7 (1)**	**No response/response**	**Weak**	**III**	**S3+**	**No**	**+**
**4**	**F**	**42**	**5**	**3**	**14**	**11 (2)**	**No response/response**	**Weak**	**III**	**S3+**	**No**	**+**
**5**	**F**	**53**	**5**	**3**	**15**	**8 (1)**	**No response/no response**	**Weak**	**III**	**S3+**	**No**	**+**
**6**	**F**	**24**	**6**	**3**	**9**	**9 (1)**	**Response/response**	**Weak**	**II**	**S3+**	**No**	**+**
**7**	**F**	**21**	**8**	**3**	**6**	**8 (1)**	**No response/response**	**Weak**	**III**	**S4**	**No**	**+**
**8**	**F**	**30**	**8**	**3**	**> 20**	**10 (1)**	**Response/response**	**No response**	**III**	**S3**	**No**	**+**
**9**	**F**	**31**	**18**	**3**	**11**	**8 (1)**	**No response/response**	**Weak**	**III**	**S3+**	**Sour, bitter**	**+**
**10**	**M**	**33**	**96**	**3**	**11**	**6 (1)**	**Response/response**	**Weak**	**II**	**S3+**	**Sweet**	**+**

Gender: F means female and M means man. Brush: Brushstroke directional sensation; horizontal, vertical, and rotational stimulating movement were applied (0 means recognized not at all, 1 means recognized only 1 direction, 2 means recognized 2 directions and 3 means recognized all movements)

2PD: Static 2-point discrimination; the values in parentheses are the opposite and control values

SWM: The Semmens-Weinstein monofilament is composed of 20 different diameter monofilaments. 1 was assigned to the smallest-diameter and 20 was the largest-diameter monofilament. The values in parentheses are the opposite and control values

Thermal: Thermal discrimination is hot and cold sensations; hot water 42 °C: cold sensation; fragment of ice 0 °C

Pin prick: Sharp touch with needle. *MRCS* Medical research Council Scale. *FSR* functional sensory recovery

**Table 5:** Postoperavive data of group A (recoded at 12 months)

No.	Age (years)	Gender	Time from injury to repair(months)	Brush	2PD (mm)	SWM	SWM (hot/cold)	Pin prick	Sunderland grade	MRCS	Gustatory sensation	FSR
**1**	**22**	**M**	**4**	**3**	**7**	**1 (1)**	**Response/response**	**Clear**	**I**	**S3+**	**Salt, Sour, Bitter, Sweet**	**+**
**2**	**34**	**M**	**4**	**3**	**10**	**8 (1)**	**Response/response**	**Clear**	**II**	**S3+**	**No**	**+**
**3**	**36**	**M**	**4**	**3**	**12**	**5 (1)**	**Response/response**	**Clear**	**II**	**S3+**	**No**	**+**
**4**	**38**	**M**	**4**	**3**	**18**	**8 (1)**	**Response/response**	**Clear**	**II**	**S3**	**salt**	**+**
**5**	**47**	**F**	**5**	**3**	**12**	**6 (1)**	**No response/response**	**Clear**	**III**	**S3+**	**No**	**+**
**6**	**30**	**M**	**6**	**3**	**13**	**4 (1)**	**Response/response**	**Clear**	**II**	**S3+**	**No**	**+**
**7**	**34**	**F**	**7**	**3**	**10**	**3 (1)**	**Response/response**	**Weak**	**II**	**S3+**	**No**	**+**
**8**	**50**	**F**	**10**	**3**	**8**	**4 (1)**	**Response/response**	**Clear**	**II**	**S3+**	**Salt, Sour, Bitter, Sweet**	**+**
**9**	**48**	**M**	**65**	**3**	**12**	**5 (1)**	**Response/response**	**Clear**	**II**	**S3+**	**No**	**+**
**10**	**35**	**F**	**90**	**3**	**15**	**5 (1)**	**Response/response**	**Clear**	**II**	**S3**	**Salt, Sour, Bitter, Sweet**	**+**

Gender: F means female and M means man. Brush: Brushstroke directional sensation; horizontal, vertical, and rotational stimulating movement were applied (0 means recognized not at all, 1 means recognized only 1 direction, 2 means recognized 2 directions and 3 means recognized all movements)

2PD: Static 2-point discrimination; the values in parentheses are the opposite and control values

SWM: The Semmens-Weinstein monofilament is composed of 20 different diameter monofilaments. 1 was assigned to the smallest-diameter and 20 was the largest-diameter monofilament. The values in parentheses are the opposite and control values

Thermal: Thermal discrimination is hot and cold sensations; hot water 42 °C: cold sensation; fragment of ice 0 °C

Pin prick: Sharp touch with needle. *MRCS* Medical research Council Scale. *FSR* functional sensory recovery

**Table 6:** Postoperavive data of group B (recoded at 12 months)

No.	Age (years)	Gender	Time from injury to repair(month)	Brush	2PD (mm)	SWM	Themal (hot/cold)	Pin prick	Sunderland grade	MRCS	Gustatory sensation	FSR
**1**	**33**	**F**	**4**	**3**	**5**	**5 (1)**	**Response/response**	**Clear**	**I**	**S4**	**No**	**+**
**2**	**36**	**F**	**5**	**3**	**10**	**6 (1)**	**Response/response**	**Weak**	**II**	**S3+**	**Salt**	**+**
**3**	**40**	**F**	**5**	**3**	**8**	**6 (1)**	**Response/response**	**Clear**	**II**	**S3+**	**No**	**+**
**4**	**42**	**F**	**5**	**3**	**14**	**10 (1)**	**No response/response**	**Weak**	**III**	**S3+**	**No**	**+**
**5**	**53**	**F**	**5**	**3**	**10**	**4 (1)**	**No response/response**	**Weak**	**III**	**S3+**	**No**	**+**
**6**	**24**	**F**	**6**	**3**	**9**	**8 (1)**	**Response/response**	**Weak**	**II**	**S3+**	**No**	**+**
**7**	**21**	**F**	**8**	**3**	**6**	**7 (1)**	**Response/response**	**Clear**	**II**	**S4**	**Salt, Sweet, Sour, Bitter**	**+**
**8**	**30**	**F**	**8**	**3**	**16**	**10 (1)**	**Response/response**	**Weak**	**II**	**S3**	**No**	**+**
**9**	**31**	**F**	**18**	**3**	**10**	**5 (1)**	**Response/response**	**Weak**	**II**	**S3+**	**Salt, Bitter**	**+**
**10**	**33**	**M**	**96**	**3**	**8**	**3 (1)**	**Response/response**	**Clear**	**II**	**S3+**	**Salt, Sweet, Sour, Bitter**	**+**

Gender: F means female and M means man. Brush: Brushstroke directional sensation; horizontal, vertical, and rotational stimulating movement were applied (0 means recognized not at all, 1 means recognized only 1 direction, 2 means recognized 2 directions and 3 means recognized all movements)

2PD: Static 2-point discrimination; the values in parentheses are the opposite and control values

SWM: The Semmens-Weinstein monofilament is composed of 20 different diameter monofilaments. 1 was assigned to the smallest-diameter and 20 was the largest-diameter monofilament. The values in parentheses are the opposite and control values

Thermal: Thermal discrimination is hot and cold sensations; hot water 42 °C: cold sensation; fragment of ice 0 °C

Pin prick: Sharp touch with needle. *MRCS* Medical research Council Scale. *FSR* functional sensory recovery

**Table 7:** Patient characteristics

	Group A (direct suturing)	Group B (collgen graft)	*P* value
Median duration from injury to repair (months)	19.9	16	0.44
Age (years)	37.4	34.3	0.375
Allodynia appearance (%)	20	20	1
Median operating time (minutes)	416	290	0.0148

*P* values were calculated using the Mann-Whitney test

Between groups A and B, there was a significant difference in median operating time

**Table 8:**
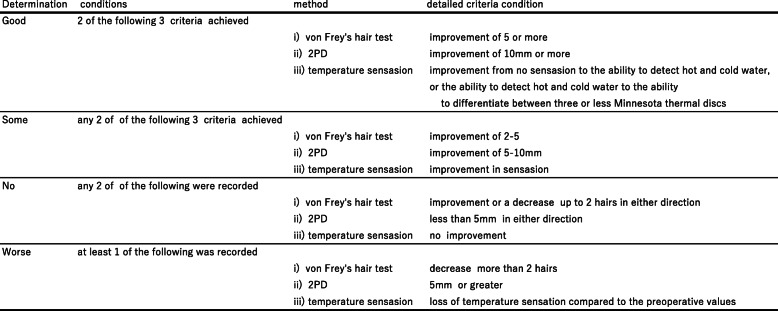
Pogrel’s postsurgical criteria

**Table 9:** Postsurgical outcomes classified by the criteria of Pogrel

	Group A (direct suturing)		Group B(collgen graft)	
	6 months	12 months	6 months	12 months
No improvement	0	0	0	0
Some improvement	8	7	7	3
Good improvement	2	3	3	7

*P* values were calculated using Fisher’s exact test

Between groups A and B at 6 and 12 months after the operation, there was always no significant difference

**Table 10:** Sunderland grades

	Group A (direct suturing)			Group B (collgen graft)		
	Preoperation	6 months	12 months	Preoperation	6 months	12 months
Grade V	4	0	0	4	0	0
Grade VI	6	0	0	6	0	0
Grade III	0	3	1	0	7	2
Grade II	0	6	8	0	3	7
Grade I	0	1	1	0	0	1

*P* values were calculated using Fisher’s exact test

Between groups A and B at preoperation and 6 and 12 months after the operation, there was always no significant difference

**Table 11:** MRCS grades

	Group A (direct suturing)			Group B (collgen graft)		
	Preoperation	6 months	12 months	Preoperation	6 months	12 months
S0	2	0	0	4	0	0
S2	8	0	0	6	0	0
S3	0	3	2	0	1	1
S3+	0	7	8	0	8	7
S4	0	0	0	0	1	2

*P* values were calculated using Fisher’s exact test

Between groups A and B at preoperation and 6 and 12 months after the operation, there was always no significant difference

## Discussion

We did not find any reports on studies comparing the outcomes between a direct perineurial suture repair and an indirect collagen allograft repair for LN injuries. Therefore, the purpose of the present investigation was to report the 1-year outcomes of both repair procedures for these injuries. Our findings revealed that there were no significant differences between groups A and B, except for the operation time. Pogrel suggested that direct repair allowed a greater improvement in the outcomes as compared to graft repair [12]. However, it is important to note that Pogrel considered only one suturing site for the direct suturing method, but two different suturing sites for the indirect collagen allograft repair. Based on our findings, we believe that other important factors may have been missed from their analysis. Conversely, Miloro proposed tension-free anastomosis and stated that allowing space to regenerate the nerve stump is important for regenerating the peripheral nerve [8]. We agree with their opinion and could reject the hypothesis that compared with the direct suture method, interpositional collagen allograft nerve repair (which simply involves two nerve anastomotic sites) has poorer outcome. We observed that irrespective of the repair method used, excellent results could be obtained by retaining space for normal peripheral nerve stumps to regenerate without interruption. In group A, a piece of the external jugular vein was utilized as a cuff; this procedure might leave a small scar in the cervical neck region and may not be preferred by young women for esthetic reasons. However, this vein collection technique is economical. Conversely, in group B, although the cost burden of the artificial material increased, the physical burden on the patient could be reduced, because the operation time in this group was shorter than the operation time in group A. There are still no global standards for the timing of an LN microsurgery repair. Bagheri et al. reported that performing a microsurgical LN repair within 9–12 months of an LN injury offered the best chance of a successful restoration of acceptable neurosensory function [12, 13]. However, Robinson and Smith found no relationship between sensory test results and a delay before microsurgical repair [14]. In our study, there were four patients in whom the time from an LN injury to repair was more than 18 months; they all experienced excellent outcomes, irrespective of the repair type. In fact, gustatory sensations had recovered even after a late LN repair in these cases. Therefore, it is considered that the criteria for LN recovery are not limited to the timing of the LN microsurgery repair. Nakanishi et al. reported that a microneurosurgery performed more than 6 months after a LN injury did not lead to a decreased recovery ratio of the sensory and taste functions; however, they noted that it did lead to a prolonged recovery of taste. This delay may be associated with a decrease in the Schwann cells in traumatic neuromas [15]. Atkins also found the criteria for LN recovery did not relate both the patient's age and a delay before microsurgical repair based on the 114 cases analysis he experienced [16]. Whenever the damaged and degenerated nerve stump can be removed under a microscopic view strictly, regardless of the time elapsed since the injury and irrespective of the repair type, we can reserve space to regenerate the nerve stump definitely.

## Conclusion

There was no significant difference in the outcomes between the two repair methods except for the operation time. In addition, the outcomes of both repair methods were excellent. We believe that ensuring a place for nerve regeneration led to such good results. In addition, excellent outcomes were achieved in four patients in whom the time from injury to repair was more than 18 months; in fact, gustatory sensations recovered even when the LN repair was performed late.

## Data Availability

Please contact the author for data requests.

## Abbreviations

LN: Lingual nerve
2PD: Two-point discrimination
FRS: Functional sensory recovery
SWM: Semmens-Weinstein monofilament
MRCS: Medical Research Council Scale
